# 25-year retrospective longitudinal study on seasonal allergic rhinitis associations with air temperature in general practice

**DOI:** 10.1038/s41533-022-00319-2

**Published:** 2022-12-06

**Authors:** Wendy Schreurs, Tjard Ronald Jacob Schermer, Reinier Peter Akkermans, Erik Wiely Maria Anton Bischoff, Hilde Dymphna Luijks

**Affiliations:** 1 Resident, Radboud Institute for Health Sciences, Department of Primary and Community Care, Radboud University Medical Centre, Nijmegen, The Netherlands; 2 Epidemiologist, Radboud Institute for Health Sciences, Department of Primary and Community Care, Radboud University Medical Centre, Nijmegen, The Netherlands; 3 Statistician, Radboud University Medical Centre, Radboud Institute for Health Sciences, IQ Healthcare, Nijmegen, The Netherlands; 4 General practitioner, Radboud Institute for Health Sciences, Department of Primary and Community Care, Radboud University Medical Centre, Nijmegen, The Netherlands

**Keywords:** Public health, Respiratory tract diseases, Medical research

## Abstract

Due to climate change, air temperature in the Netherlands has gradually increased. Higher temperatures lead to longer pollen seasons. Possible relations between air temperature and increased impact of seasonal allergic rhinitis (SAR) in general practice have not been investigated yet. We explored trends in timing of frequent seasonal allergic rhinitis presentation to general practitioners (GPs) over 25 years and explored associations with air temperature. We performed a retrospective exploratory longitudinal study with data from our Family Medicine Network (1995–2019), including all SAR patients and their GP-encounters per week. We determined patients’ GP-consultation frequency. Every year we identified seasonal periods with substantial increase in SAR related encounters: peak-periods. We determined start date and duration of the peak-period and assessed associations with air temperature in the beginning and throughout the year, respectively. The peak-period duration increased by a mean of 1.3 days (95% CI 0.23–2.45, *P* = 0.02) per year throughout the study period. Air temperature between February and July showed a statistically significant association with peak-period duration. We could not observe direct effects of warmer years on the start of peak-periods within distinct years (*P* = 0.06). SAR patients’ contact frequency slightly increased by 0.01 contacts per year (95% CI 0.002–0.017, *P* = 0.015). These longitudinal findings may help to facilitate further research on the impact of climate change, and raise awareness of the tangible impact of climate change in general practice.

## Introduction

Allergic rhinitis is a common condition, caused by inflammation of the nasal mucosa after sensitisation to (mostly) aeroallergens. Symptoms are sneezing, rhinorrhoea, nasal obstruction and an itch in the nose and throat^1^.

Allergic rhinitis may also cause more general symptoms such as fatigue, malaise and sleep disturbance and can have a negative influence on daily functioning^2–4^.

Patients experience decreased work productivity and sick leave with costs estimated to be €961 per individual/year for the Swedish population^5^. Seasonal allergic rhinitis (SAR) is allergic rhinitis with seasonal symptoms, caused by inhaled grass or tree pollen. Many patients with SAR are polysensitised and experience symptoms year-round^2^. Symptoms are similar to those of ‘perennial’ AR caused mainly by indoor aeroallergens (house dust mites, animal dander).

The prevalence of recorded allergic rhinitis in health records in general practice ranges from 0.4 to 4.1%, but the discrepancy with prevalence of self-reported symptoms suggests (substantial) underreporting of allergic rhinitis in general practice databases because those with mild disease do not attend the GP but go to the pharmacy^2,6,7^.

In the period 1951–2013, the average air temperature in the Netherlands has increased by 1.4 degrees Celsius and is expected to rise even further in future years^8^. The start of the pollen season depends on various factors, including air temperature. There is now substantial evidence that increasing temperature influences the timing of flowering and the start, peak, end and magnitude of pollen seasons in different climate zones^9–12^.

Pollen developing at higher temperatures or in polluted air have a stronger allergenicity^13^. Climate change introduces new plant species in regions that are able to survive in higher temperatures, including allergenic species^14^. In addition, warmer temperatures in winter or early spring lead to less changes in flowering and leafing onsets of plants, and consequently to an earlier start of the pollen season^15^. A warm end of the year causes plants to flower longer or to flower again^14^. Finally, air pollution may influence pollen allergenicity. An increase in temperature thus results in a longer pollen season. This may lead to an extension of the period in which patients with SAR experience and present symptoms. To date, clinical studies that investigate this relation are lacking^16^.

What previous studies have shown is an increased risk for developing SAR after exposure to extreme heat events^17^. In a South Korean study, associations between air temperature in March, aerial pollen concentrations, and hospital visits for SAR were studied. The investigators found an association between higher minimum temperature in March and increased hospital visits from April to July^18^.

Despite a growing global engagement with climate change, including specifically among health professionals^19,20^, little is known about the tangible impact of climate change on general practice. We aimed to investigate the association between patient consultation rates in general practice for SAR complaints and air temperature over a substantial time period. In the Netherlands, where the initial contact with the healthcare system is through the GP, consistent, longitudinal, accurate morbidity recording in Practice-Based Research Networks (PBRNs) enables studying GP encounters for SAR in retrospect. In the Netherlands, GPs are the health professionals with complete overview of their patients’ (known) health problems since all initial contacts with the healthcare system occur through the GP. We hypothesised that increased temperature led to a greater impact of SAR on general practice: [1] a warmer start of the year is associated with an earlier start of presented SAR symptoms to GPs; [2] warmer years in general are associated with a longer duration of the period with symptomatic SAR presentation to GPs; and [3] increased temperature is associated with increased severity of SAR, resulting in a higher frequency of SAR-related contacts with GPs over time.

## Results

### Patient characteristics and descriptive data

Table 1 shows the characteristics of the included SAR patients and the total patient population of five single years during the study period (not all years are presented). It consistently shows a larger proportion of females among the SAR patients. The recorded prevalence of SAR throughout our study period ranged from 17.6 (in 1995) to 58.4 per 1000 patient years (in 2012).

**Table 1:** Characteristics of SAR patients and the total patient population within the general practices of some of the years.

Characteristics per year^a^	1995^b^	2000	2005	2010	2015	2019^b^
Total patient years of the general practices	6526	6294	6961	7326	9783	4066
SAR prevalence per 1000 patient years^c^	17.6	38.1	41.5	51.5	38.6	28.5
SAR patient years	115	240	289	377	378	186
% Female	63.5%	64.6%	57.8%	56.8%	56.3%	59.1%
Mean age of SAR patients (in years)^d^	41 (SD 20.1)	37 (SD 20.0)	39 (SD 20.1)	40 (SD 21.2)	40 (SD 21.6)	46 (SD 20.1)
Mean age among males (in years)	36 (SD 21.4)	**32 (SD** 20**.7)**	35 (SD 21.7)	34 (SD 20.2)	38 (SD 22.5)	43 (SD 21.5)
Mean age among females (in years)	43 (SD 20.4)	40 (SD 19.0)	41 (SD 18.5)	44 (SD 20.9)	42 (SD 20.8)	48 (SD 19.0)

^a^Single years data—not all years are presented.

^b^In 1995, only practice 1 and 2 are included because practice 3 started registering in 1996. Practice 1 stopped registering in 2017 and does not contribute to the data in 2019.

^c^Prevalence per 1000 patient years is calculated as the proportion of SAR patient years and total patient years of the general practice.

^d^Mean age is calculated at the end of the year.

### Trends in temperature and peak-period over time

The mean annual CNT showed a significant increase of 0.041 degrees Celsius every year during our study period (95% CI 0.004–0.077, *P* = 0.029).

For every observation year, the peak-period started between week 8 (i.e., end of February) and 19 (beginning of May). The week in which the peak-period started showed a significant linear trend over time, starting 1.7 day earlier per subsequent year (95% CI 0.65–2.73, *P* = 0.003). Moreover, the duration of the peak-period significantly increased by 1.3 days per year (95% CI 0.23–2.45, *P* = 0.02). Over the first and last five years of the study period (i.e. 1995–1999 and 2015–2019) we calculated averages for start and for duration of the peak period. Peak-periods started in week 15/16 and lasted 98 days on average in 1995–1999. Start and average duration in 2015–2019 were week 11/12 and 122 days, respectively. The ending of the peak-period fell in July (i.e., between week 27 and 31) and showed no statistically significant changes over time (−0.35 days per year, 95% CI −0.85–1.54, *P* = 0.17).

The SAR contact frequency showed a small, statistically significant increase of 0.01 encounters per year over time (95% CI 0.002–0.017, *P* = 0.015). The mean number of encounters per patient year increased from 2.0 over the first 5 years of the study period (i.e. 1995–1999) to a mean of 2.2 over the last 5 years (2015–2019).

### Association between air temperature and SAR encounters

With regard to hypothesis 1, the start of the peak-period was earlier for the years with a relatively warm March than for the other years (Table 2). The 75th percentile of the week in which the peak-period started in warmer years (i.e., the warm years with a relatively late start of the peak-period) seemed to fall earlier than the 25th percentile (a relatively early start of the peak) in the colder years. There was weak evidence of an association between warm temperatures in March and an early start of the peak-period (*P* = 0.06, Table 2). Performing the same analysis with the CNT of January or February did not show (statistically significant) associations.

**Table 2:** Differences in timing of the start of the SAR peak period between relatively cold or warm start of the year.

	Start of the peak period (in week number)				
	Q1	Median	Q3	Mean Rank Score	Kruskal–Wallis *P* value
CNT in January					0.44
Cold	13	15	16	15.6	
Intermediate	10	14	15	11.3	
Warm	12	14	16	14.2	
CNT in February					0.70
Cold	10	13	15	11.0	
Intermediate	12	14	16	14.0	
Warm	12	14–15	15	12.8	
CNT in March					0.06
Cold	15	15–16	17	17.6	
Intermediate	12	14	16	13.4	
Warm	8	11–12	13	7.5	

Cold is defined as CNT < Q1 (=relatively low CNT).

Intermediate is defined as CNT between Q1 and Q3 (=median CNT).

Warm is defined as CNT > Q3 (=relatively high CNT).

Q1 = 1.79 degrees Celsius. Median = 4.33 degrees Celsius. Q3 = 5.86 degrees Celcius.

With regard to hypothesis 2, Table 3 shows the temperature throughout the first half of the year (cold, intermediate, warm) related to the duration of the SAR peak-period. The duration of the peak-period was longer in the groups with a cold and a warm average temperature over the period February–July (*P* = 0.02). No difference in peak-period duration was observed in the analyses with the CNT of February till May or February till June.

**Table 3:** Differences in length of the SAR peak period between relatively cold or warm periods of time.

	Length of the peak period (in weeks)				
	Q1	Median	Q3	Mean Rank score	Kruskal–Wallis *P* value
CNT in February till May					0.50
Cold	14.0	16.5	19.0	15.8	
Intermediate	14.0	15.0	17.0	12.6	
Warm	12.0	14.0	17.0	11.1	
CNT in February till June					0.40
Cold	14.0	16.5	19.0	15.8	
Intermediate	14.0	15.0	15.0	11.2	
Warm	13.0	16.0	19.0	14.1	
CNT in February till July					**0.02**
Cold	15.0	18.5	19.0	18.3	
Intermediate	12.0	14.0	15.0	9.1	
Warm	15.0	16.0	19.0	16.3	

Cold period is defined as CNT < Q1 (=relatively low CNT).

Intermediate period is defined as CNT between Q1 and Q3 (=median CNT).

Warm period is defined as CNT > Q3 (=relatively high CNT).

Q1 = 7.88 degrees Celsius. Median = 8.43 degrees Celsius. Q3 = 9.22 degrees Celcius.

Bold value indicates statistical significant *P* value (*P* < 0.05).

## Discussion

Urged by global warming and evidence of prolonged pollen seasons, we performed the first clinical study investigating the relation between air temperature and symptomatic SAR in general practice. In an exploratory longitudinal study based on 25 years of robust general practice data we examined trends over time of the start and length of peaks in presentation of SAR symptoms. We found an earlier start (1.7 days/year) and prolonged duration (+1.3 days/year) of the SAR season over time, and a modest increase in contact frequency of SAR patients with their GP over time (+0.25 encounters over 25 years). Statistical testing showed a significant association between temperature from February until July and the duration of the SAR peak, although direct effects of a warmer start of distinct years on the timing of the start of the peak in this particular year were not observed (*P* = 0.06 for a warm March).

A major strength of this study is the data source: FaMe-Net is a reliable practice-based research network representative for the Dutch population regarding age, sex and social class^21,22^. It is the world’s longest uninterrupted primary care morbidity registration, unique in using an unaltered disease classification system over this long period of time^21,22^, which enabled us to study 25 years of general practice data in retrospect—starting long before climate change awareness raised. Although FaMe-Net’s population size is substantial, only large effects of temperature on the outcome measures could be shown conclusively, since each calendar year included counted as a single observation in the statistical models. Despite the robust dataset, this resulted in limited statistical power for our study. It’s questionable if the three practices we included are representative to all Dutch general practices. We tried to include more practices, but this introduced bias due to different follow-up periods between the practices.

SAR is considered a chronic disease, but the burden patients experience can alter over time^23^. We defined ‘SAR patients’ as patients contacting the GP for SAR within the particular year (contact prevalence proportion)^24^. This may have led to fluctuations in our recorded prevalence of SAR. The logical alternative, considering SAR as chronic disease after a patient’s initial diagnosis onwards, likely creates overestimation of clinically relevant SAR in an ageing population within our long study period. To increase internal validity, we included only the practices that continuously and uniformly registered during our entire study period of 25 years. To minimise potential bias due to a changing prevalence over the years we corrected our outcome measures by dividing by the number of SAR ‘patient years’. Obviously, only morbidity that patients present to their GP can be recorded so that it reflects a proportion of perceived symptoms. Other potential biases such as changes in consultation behaviour may also remain. Moreover, increased accessibility of SAR medication during our study period may have resulted in an underestimation of the increased health care demand (+0.25 encounters per SAR patient per year over 25 years)^25^.

Finally, misclassification might have played a role. Perennial and seasonal allergic rhinitis may co-occur as do allergic and non-allergic rhinitis^26^. Thanks to the ICD-10 subcoding in FaMe-Net we were able to exclude isolated registered ‘perennial AR’. However, it is not possible to register multiple specific subcodes of ‘allergic rhinitis’. This might have resulted in excluding SAR patients with predominant ‘other specific allergy’. On the other hand, presented ‘other’ allergic symptoms (other than specific ‘SAR’) of included ‘SAR patients’ may have been counted as ‘SAR’ encounters. This potential misclassification might have introduced bias, in either direction. We expect that such bias in two possible directions will mediate and has not had relevant impact on our results.

We explored a new and reproducible method to identify SAR peak-periods, but it might be influenced by extreme values. We limited the potential effects of extreme values by basing the cut-off point on the mean of all 25 years. Besides temperature, other factors, e.g. rainfall^9^, traffic-related pollutants^27,28^ and level of urbanisation^27,29^ may also influence the timing of the start and length of the SAR season and its severity, since they influence local pollen concentrations. We had no data on these potential confounders. In some years, we found more than one peak leading to a possible overestimation of the whole peak period within these particular years. It could have been helpful to couple these peaks to pollen concentrations to distinguish between peaks caused by tree and/or grass pollen. Unfortunately, data on pollen concentrations were not available for our study. Finally, local variations in contributing factors (e.g. temperature) might have played a role, but we were unable to determine them since our calculations were based on the CNT. Further research could relate clinical data to local factors (local temperature, pollen concentrations, level of urbanisation/pollution) and strengthen our results.

We interpret the observed increased frequency of SAR over time as increased severity of SAR. This corresponds with increasing prevalence of SAR after heat exposure^17^. A previous study reported more GP visits among patients with moderate/severe SAR compared to patients with mild disease^28^. Our findings are in line with the only previous study we identified that related temperature and timing of SAR symptoms. Kim and colleagues studied hospital visits as primary outcome and found that a high minimum temperature in March was positively correlated with the number of hospital visits by SAR patients from April to July^18^. Our primary care results suggest that a warm month of March could lead to an earlier start of the SAR season. The association we observed, however, did not reach statistical significance (*P* = 0.06).

This study derived presence of SAR symptoms from longitudinally registered GP encounters for SAR. Although GPs only see 1–2% of all patients with SAR symptoms^30^, these patients still lead to a substantial demand of health care. The recorded prevalence and the contact frequency of SAR patients with their GP we observed in our study correspond with those reported in another large Dutch primary care dataset^31^.

Apart from SAR, other respiratory conditions also seem to be influenced by climate change. Air pollution increases the incidence and severity of upper respiratory tract infections, and aggravates asthma. Interior warming of houses stimulates proliferation of allergens inside homes such as dust mites and fung^32^. As goes for SAR, the evidence could be strengthened to demonstrate that climate change indeed negatively impacts these other conditions.

The increased contact frequency for SAR we found over time [hypothesis 3] may reflect increased severity of SAR. We consider the increased mean consultation from 2.0 (1995–1999) to 2.2 encounters per year (2015–2019) clinically relevant, especially given the increased availability of SAR medication without consulting a doctor, and due to increased access to health education, e.g. thanks to patient education website GPinfo (www.thuisarts.nl) launched in 2011.

A low or high average temperature from February till July resulted in a significantly longer duration of the SAR season. This result can be partly explained: a higher temperature leads to earlier spring, and thereby earlier start of the (tree) pollen season and probably a longer SAR season^33^. A lower temperature results in more simultaneous blooming, which seems to affect pollen concentrations, causing more severe SAR complaints^34^. Polysensitisation probably contributes^4^. We can only speculate if a strong initial response somehow triggers sustained complaints, explaining the longer duration of the SAR peak.

These signs of increased severity of SAR (more contacts per year per SAR patient), and the prolongation of the period of the year in which patients present to GPs with symptoms of SAR—including a tendency towards earlier start of SAR seasons—are likely caused by climate change. They result in a higher burden for patients and a higher workload for GPs, and hence are important to patients, primary care clinicians and policy makers.

However, our study had limited statistical power. Therefore additional and larger scale research is needed. The methods we applied could be easily reproduced by other Practice-Based Research Networks that record data on SAR in different regions so that data could be combined.

Data from weather stations from different countries and regions could help to study the association between SAR symptoms and temperature more precisely.

Collaboration between clinicians and climate scientists or biologists is needed to make more direct links between clinical and environmental data, by addition of pollen concentrations, frost-free days and cumulative degree days. For patients to self-manage SAR, and to predict impact on medical services, it could be helpful to provide accurate forecasts. This requires local instead of regional data which is not always available, let alone data of all factors that can influence the pollen season^35^. Our current study might help to find a method to eventually forecast the pollen season without acquiring to much environmental data.

Climate change has other health effects besides respiratory problems. Heat waves result in heat strokes, renal function problems and cardiovascular disease. Ultraviolet radiation increases skin cancer risk. Additionally, it results in more infections transmitted by water, food and vectors^32^.

Globally, awareness of the climate crisis and the need for urgent action has massively increased over the last few years. Linking climate change to adverse health outcomes probably helps to make the need to respond to this crisis urgently recognisable for more individual people. The health profession has an obvious role in identifying such associations. Just as happened in the past regarding advancements in sanitation, hygiene and tobacco control, health professionals should and are indeed showing responsibility and leadership^19,20^.

In this first study linking air temperature to clinical presentation of SAR to GPs, our hypotheses that increased temperature led to a greater impact of SAR on general practice were confirmed: we observed a longer duration of the period in which SAR was presented, with a tendency towards an earlier start of SAR presentation in warmer years, and increased frequency of GP contacts for SAR, suggesting increased severity. Statistical power of this study was limited, justifying careful interpretation. Evidence directly linking climate change to adverse health outcomes is still limited but steadily growing in the past few years. Our results call for additional research to confirm and strengthen our findings, for which our exploratory methods can serve as an example for other PBRNs.

## Methods

### Setting

This study is a retrospective exploratory longitudinal study, using data from 1995 up to and including 2019 from the Dutch Family Medicine Network (FaMe-Net) database. This PBRN resulted from a fusion in 2013 of two predecessor networks. To the best of our knowledge this is the PBRN with the longest uninterrupted registration period worldwide^29^. Due to its position embedded in the Dutch healthcare system, FaMe-Net GPs have a complete overview of all health problems of listed patients, including specialist reports and out-of-hours general practice care. The network currently consists of six general practices providing regular primary care to their patients and is representative for the Dutch population^29^. The FaMe-Net registration for research purposes occurs during daily practice, is reliable, and has resulted in numerous publications (http://www.famenet.nl)^21,22^. All morbidity is carefully coded according to the *International Classification of Primary Care* (ICPC-2) and is unique in additionally assigning *International Classification of Diseases and Related Health Problems* (ICD-10) subcodes to all diagnoses. Registration is ‘episode-based’, which means that all information belonging to the same health problem is ordered in one ‘episode of care’ and can be traced back. The network remained stable over time but some practices have joined or left the network. Therefore not all participating practices consistently contributed data over the entire study period.

To ensure high consistency between practices, we decided to include only the three practices that registered with the same classification system during the entire study period (1995–2019). Two practices are small practices (<3000 patients) located in the west of the Netherlands and the other is a medium size practice (3000–6000 patients) located in the north (http://www.famenet.nl).

Air temperature data were derived from the Royal Netherlands Meteorological Institute (KNMI). We used the Central Netherlands Temperature (CNT) data which is especially constructed for climate models and represents the average temperature in the area between the Dutch cities of Utrecht, Arnhem, Breda and Eindhoven (https://www.knmi.nl/kennis-en-datacentrum/achtergrond/centraal-nederland-temperatuur-cnt). The CNT is constructed based on monthly average temperature series of six weather stations in the Netherlands.

We investigated our hypotheses by relating [1] the start of the peak-period (early/late) to the CNT of January, February and March (categorised as warm, intermediate or cold), [2] the duration of the peak-period (long/short) to the CNT of February till May, February till June, and February till July (warm/intermediate/cold), and finally, as additional outcome [3] by testing a potential increase in the number of SAR related encounters per SAR patient over time.

### Patient population

We included all patients who were registered with one or more episodes of care for ‘SAR’, classified with ICPC code R97 “allergic rhinitis” or F71 “allergic conjunctivitis” between 1 January 1995 and 31 December 2019. The ICD-10 subcoding with J30.3 “other specified allergic rhinitis” enabled exclusion of patients with another specific allergy instead of SAR, e.g. allergy for cats, dogs or house dust mites. Patients with this ICD-10 subcode, and patients with an ICPC code R97 who had a negative specific IgE test result (previously: radioallergosorbent test, RAST) result for tree and grass pollen, but a positive RAST for dust mites, cats or dogs allergens, were excluded.

### Outcome measures

SAR symptoms are dependent on the local aerial pollen concentration and are therefore seasonal. This is reflected in week-by-week variability in SAR related encounters in general practice. The SAR related encounters were all office, telephone and email GP consultations, repeat prescriptions and home visits, as registered by the GPs in episodes of care for SAR of the included patients.

Since we could not locate previous studies with a similar research question we had to define precisely how we assessed the start, ending and duration of ‘peak periods’. Robust exploration of visualised data (graphs) of the SAR contacts showed that an increase and decrease in contact frequency occurred suddenly every year. We formulated mathematical rules defining the start, ending and duration of peak periods (Box 1). The beginning [hypothesis 1] and the duration [hypothesis 2] in weeks of the annual peak-period of SAR related encounters served as the primary outcome measures for this study, supported by literature comparing these items to the CNT^14–16,25^. Every year is one outcome value, resulting in *n* = 25 years to be studied (1995 up to and including 2019). Since the literature does not provide clear guidance on the exact length of the period to study for CNT, we defined multiple periods differing in duration, over which the mean temperature was calculated, i.e. February until May, February until June, and February until July. These periods were categorised with low, median or high average temperature as occurring in the CNT.

To investigate a possible higher workload for GPs, our additional outcome measure was the ‘SAR contact frequency’ (Box 2), which can be interpreted as a measure for the severity of SAR symptoms [hypothesis 3]. Within each observation year, it divides all ‘SAR related encounters’ by the number of ‘SAR patient years’, thus correcting for the actual population affected by SAR. This makes the start and duration of the peak period comparable for the different years. ‘SAR patient years’ were counted for ‘SAR patients’: all patients with at least one SAR-related encounter with the practice in the corresponding year and corrected for the actual time patients were listed with the practice (full years or shorter).

To define ‘peak-periods’ (Box 1), we additionally expressed the ‘SAR contact frequency’ as a weekly measure: the ‘weekly contact frequency’. The number of ‘SAR related encounters’ was assessed for each separate week number and then divided by ‘SAR patient years’ in that particular year.

Box 1: Methods to identify peak-periods Visual inspection of graphs of hay fever-related contact frequency for separate calendar years in our study period fitted well to ‘peak periods’ of hay fever as referred to in the literature. We had to operationalise a definition for these ‘peak periods’ since we did not find any in the previous literature. We formulated the following definition, which we assessed as appropriate to the visual data for all 25 included years. A peak was assigned when the weekly hay fever contact frequency was above the cut-off value* for 3 weeks in a row OR when the cut-off value was exceeded for 2 consecutive weeks, then interrupted by 1 week, followed by a series of 3 or more weeks above the cut-off value. The peak ended when two consecutive values were below the cut-off value. The cut-off value was determined as the mean of the weekly contact frequency in the months with the least allergenic pollen in the air (November, December, January). A year could contain more than one peak period. Therefore the duration of the a peak period was determined as the time from the start of the first peak until the end of the last peak of the year.

*cut-off value: the mean of the weekly contact frequency in November, December and January of all 25 years.

As an example of the visual inspection: the graphic of 2009, the begin and the end of the peak period, defined as described above, are marked with the blue arrows. (Begin starts at week 13, and end is at week 28).
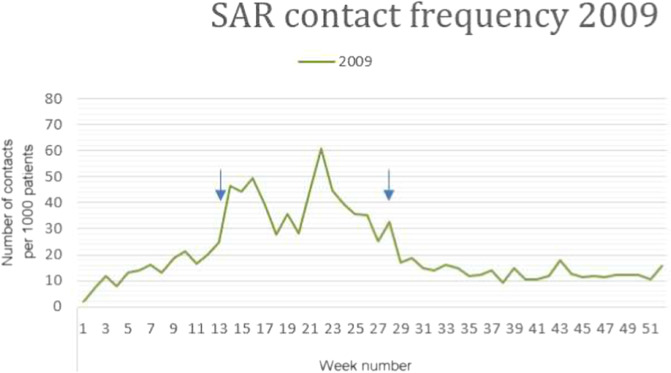

Box 2: SAR contact frequency The SAR contact frequency is used to approach the number of SAR related encounters per patient that can be interpreted as a measure for the severity of SAR symptoms. We assume that symptoms are more severe when more encounters per patient are needed. To calculate the SAR contact frequency, we used the following formula:

$${\rm{SAR}}\;{\rm{contact}}\;{\rm{frequency}} = \frac{{{\rm{Number}}\;{\rm{of}}\;{\rm{all}}\;{\rm{related}}\;{\rm{encounters}}\;{\rm{in}}\;{\rm{the}}\;{\rm{corresponding}}\;{\rm{year}}\;\left( {{\rm{for}}\;{\rm{example}}\;2015} \right)}}{{{\rm{the}}\;{\rm{number}}\;{\rm{of}}\;{\rm{SAR}}\;{\rm{patient}}\;{\rm{years}}\;{\rm{of}}\;{\rm{the}}\;{\rm{corresponding}}\;{\rm{year}}}}$$

With more encounters per patient, the workload for GPs per patient will also be higher.

### Statistical methods

We performed linear regression analyses in SPSS, version 25, to determine changes over time for the different outcome measures: [1] the start and [2] the duration of the peak-period and [3] the SAR contact frequency.

To test our hypotheses, we investigated potential differences in start [1] and duration [2] of the peak-period between years with a cold, intermediate or warm mean temperature using the Kruskal–Wallis test. Because every year contributes only one single outcome value, resulting in *n* = 25, only a large linear effect could be demonstrated when using continuous variables. This compelled us to categorise our temperature data. Scores between the 25th and 75th percentile were assigned ‘intermediate’. To focus on the outliers (warm and cold) using quartiles, we decided to create a bigger ‘mid-section’ (intermediate) group by creating three categories (not four, i.e. <25, 25–75, >75).

To test our third hypothesis, we performed a regression analysis of the contact frequency of SAR encounters divided by the number of SAR patient years over time, to study a possible increase in the incidence of SAR encounters per patient year over time. A *P* value < 0.05 was considered statistically significant.

### Ethical regulations

This study complied with all ethical regulations. The medical ethical committee of our institution approved the execution of scientific research with the existing data from the FaMe-Net network database, and waived specific approval for this particular study.

### Reporting summary

Further information on research design is available in the Nature Research Reporting Summary linked to this article.

## Supplementary information

REPORTING SUMMARY

## Data Availability

The data that support the findings of this study are available from the corresponding author upon reasonable request. This request should contain the reason and purpose for the request, and the location and the qualifications of the researcher. Researchers can submit an application to obtain the data to the FaMe-Net coordinator (currently dr. Annemarie Uijen; annemarie.uijen@rabdouumc.nl), the head of the academic general practitioners network (currently Prof. Henk Schers; Henk.Schers@radboudumc.nl) or the Radboud Technology Center Health Data (RTC.Healthdata@radboudumc.nl). They assess the relevance and reasonability of the request. The data itself contain delicate information. In consultation with Research Data Management of Radboud University, it was decided not to publish the data for privacy reasons.

## References

[1] Dykewicz MS (2020). Rhinitis 2020: a practice parameter update. J. Allergy Clin. Immunol..

[2] Brozek JL (2017). Allergic Rhinitis and its Impact on Asthma (ARIA) guidelines – 2016 revision. J. Allergy Clin. Immunol..

[3] Meltzer EO (2009). Sleep, quality of life, and productivity impact of nasal symptoms in the United States: findings from the Burden of Rhinitis in America survey. Allergy Asthma Proc..

[4] Valovirta E, Myrseth SE, Palkonen S (2008). The voice of the patients: allergic rhinitis is not a trivial disease. Curr. Opin. Allergy Clin. Immunol..

[5] Cardell LO (2016). TOTALL: high cost of allergic rhinitis—a national Swedish population-based questionnaire study. NPJ Prim. Care Respir. Med..

[6] Pols DH (2016). Atopic dermatitis, asthma and allergic rhinitis in general practice and the open population: a systematic review. Scand. J. Prim. Health Care.

[7] KNMI. (2014). Climate Change Scenarios for the 21st Century - A Netherlands Perspective.

[8] Bousquet J (2006). Severity and impairment of allergic rhinitis in patients consulting in primary care. J. Allergy Clin. Immunol..

[9] Damialis A, Smith M, Galán C (2021). Editorial: Climate change and aeroallergens. Front. Allergy.

[10] Ziska, L. H. Climate, carbon dioxide, and plant-based aero-allergens: a deeper botanical perspective. *Front. Allergy* **2**, 714724 (2021). 10.3389/falgy.2021.714724 PMC8974748 35386997

[11] De Weger, L. A. et al. Long-term pollen monitoring in the benelux: evaluation of allergenic pollen levels and temporal variations of pollen seasons. *Front. Allergy* **2**, 676176 (2021). 10.3389/falgy.2021.676176 PMC8974733 35387026

[12] Menzel, A., Ghasemifard, H., Yuan, Y. & Estrella, N. A first pre-season pollen transport climatology to Bavaria, Germany. *Front. Allergy* **2**, 627863 (2021). 10.3389/falgy.2021.627863 PMC8974717 35386987

[13] Ouyang Y, Zhaoyin Y, Li Y, Erzhong F, Zhang L (2019). Associations among air pollutants, grass pollens, and daily number of grass pollen allergen-positive patients: a longitudinal study from 2012 to 2016. Int. Forum Allergy Rhinol..

[14] Huynen, M. et al. ZonMw-Kennisagenda klimaat en gezondheid. https://www.zonmw.nl/nl/actueel/nieuws/detail/item/kennisagenda-klimaatverandering-en-gezondheid-nederland-geconfronteerd-met-groot-aantal-gezondheids/ (2019).

[15] Mehdipoor, H., Zurita-milla, R., Augustijn, E. & Van Vliet, A.J.H. Checking the consistency of volunteered phenological observations while analysing their synchrony. *ISPRS Int. J. Geo Inf*. **7**, 487 (2018).

[16] Jalbert I, Golebiowski B (2015). Environmental aeroallergens and allergic rhino-conjunctivitis. Curr. Opin. Allergy Clin. Immunol..

[17] Upperman CR (2017). Exposure to extreme heat events is associated with increased hay fever prevalence among nationally representative sample of US Adults: 1997-2013. J. Allergy Clin. Immunol..

[18] Kim SH, Park HS, Jang JY (2011). Impact of meteorological variation on hospital visits of patients with tree pollen allergy. BMC Public Health.

[19] Watts N (2021). The 2020 report of The Lancet Countdown on health and climate change: responding to converging crises. Lancet.

[20] Atwoli L (2021). Call for emergency action to limit global temperature increases, restore biodiversity, and protect health. Med. J. Aust..

[21] Luijks H (2021). Purposeful incorporation of patient narratives in the medical record in the Netherlands. J. Am. Board Fam. Med..

[22] Schers H (2021). The COVID-19 pandemic in Nijmegen, the Netherlands: changes in presented health problems and demand for primary care. Ann. Fam. Med..

[23] Nihlén U (2006). Incidence and remission of self-reported allergic rhinitis symptoms in adults. Allergy.

[24] Spronk I (2019). Calculating incidence rates and prevalence proportions: not as simple as it seems. BMC Public Health.

[25] Tan R (2017). Identifying the hidden burden of allergic rhinitis (AR) in community pharmacy: a global phenomenon. Asthma Res. Pract..

[26] Hellings PW (2017). Non-allergic rhinitis: Position paper of the European Academy of Allergy and Clinical Immunology. Allergie.

[27] Zhao F (2016). Common ragweed (*Ambrosia artemisiifolia* L.): allergenicity and molecular characterization of pollen after plant exposure to elevated NO2. Plant Cell Environ..

[28] Ghiani A, Aina R, Asero R, Bellotto E, Citterio S (2012). Ragweed pollen collected along high-traffic roads shows a higher allergenicity than pollen sampled in vegetated areas. Allergy.

[29] Price D (2015). The hidden burden of adult allergic rhinitis: UK healthcare resource utilisation survey. Clin. Transl. Allergy.

[30] Ross AM, Fleming DM (1994). Incidence of allergic rhinitis in general practice, 1981-92. BMJ.

[31] Van Dijk, C., Verheij, R. & Schellevis, R. *Hooikoorts in de Huisartsenprakijk: Kosten en Verleende Zorg* (NIVEL, 2010).

[32] Luijks H (2021). Purposeful incorporation of patient narratives in the medical record in the Netherlands. J. Am. Board Fam. Med..

[33] Sapkota, A. et al. Associations between alteration in plant phenology and hay fever prevalence among US adults: implication for changing climate. *PLoS ONE* **14**, e0212010 (2019). 10.1371/journal.pone.0212010 PMC6438449 30921361

[34] Quarsie, J., van de Pas, R., Fanoyen, E. & van den Hazel, P. De impact van klimaatverandering op gezondheid in Nederland. *Ned. Tijdschr. Geneeskund*. **165**, D6245 (2021). 34523842

[35] Kurganskiy, A. et al. Predicting the severity of the grass pollen season and the effect of climate change in Northwest Europe. *Sci. Adv*. **7**, eabd7658 (2021). 10.1126/sciadv.abd7658 PMC7997511 33771862

