# Supplementary material for: 25-year retrospective longitudinal study on seasonal allergic rhinitis associations with air temperature in general practice
Source: NPJ Prim Care Respir Med. 2022 Dec 6;32:54. doi: 10.1038/s41533-022-00319-2 (PMC9723707; doi:10.1038/s41533-022-00319-2)
Supplement: Supplementary file 1 — REPORTING SUMMARY [file 41533_2022_319_MOESM1_ESM.pdf]

nature portfolio

Corresponding author(s): Wendy Schreurs, Tjard Ronald Jacob Schermer, Reinier Peter Akkermans, Erik Wiely Maria Anton Bischoff, Hilde Dymphna Luijks.

Last updated by author(s): May 27, 2022

Reporting Summary

Nature Portfolio wishes to improve the reproducibility of the work that we publish. This form provides structure for consistency and transparency in reporting. For further information on Nature Portfolio policies, see our Editorial Policies and the Editorial Policy Checklist.

Statistics

For all statistical analyses, confirm that the following items are present in the figure legend, table legend, main text, or Methods section.

n/a	Confirmed	
☐	☒	The exact sample size (*n*) for each experimental group/condition, given as a discrete number and unit of measurement
☒	☐	A statement on whether measurements were taken from distinct samples or whether the same sample was measured repeatedly
☐	☒	The statistical test(s) used AND whether they are one- or two-sided<br>*Only common tests should be described solely by name; describe more complex techniques in the Methods section.*
☐	☒	A description of all covariates tested
☐	☒	A description of any assumptions or corrections, such as tests of normality and adjustment for multiple comparisons
☐	☒	A full description of the statistical parameters including central tendency (e.g. means) or other basic estimates (e.g. regression coefficient) AND variation (e.g. standard deviation) or associated estimates of uncertainty (e.g. confidence intervals)
☐	☒	For null hypothesis testing, the test statistic (e.g. *F*, *t*, *r*) with confidence intervals, effect sizes, degrees of freedom and *P* value noted<br>*Give P values as exact values whenever suitable.*
☒	☐	For Bayesian analysis, information on the choice of priors and Markov chain Monte Carlo settings
☒	☐	For hierarchical and complex designs, identification of the appropriate level for tests and full reporting of outcomes
☒	☐	Estimates of effect sizes (e.g. Cohen's *d*, Pearson's *r*), indicating how they were calculated

Our web collection on statistics for biologists contains articles on many of the points above.

Software and code

Policy information about availability of computer code

Data collection	Registration of data on diagnosis and contact frequency for SAR occurs within the electronic health record (EHR) in the general practice. Registering practices are affiliated to the practice based research network (PBRN) which is a collaboration between the practices and the Radboud University Medical Centre. All data registered for the PBRN are extracted from the EHR and stored de-identified in the Radboudumc Technology Center (RTC) Health Data. RTC processes and secures the data for research purposes and secures privacy of involved patients. Listed patients in the practices may opt-out for extraction of their data. This procedure complies with all Dutch privacy legislation.
Data analysis	SPSS software, version 25.

For manuscripts utilizing custom algorithms or software that are central to the research but not yet described in published literature, software must be made available to editors and reviewers. We strongly encourage code deposition in a community repository (e.g. GitHub). See the Nature Portfolio guidelines for submitting code & software for further information.

Data

Policy information about availability of data

All manuscripts must include a data availability statement. This statement should provide the following information, where applicable:

- Accession codes, unique identifiers, or web links for publicly available datasets
- A description of any restrictions on data availability
- For clinical datasets or third party data, please ensure that the statement adheres to our policy

The datasets analysed during the current study are available from the corresponding author on reasonable request.

Human research participants

Policy information about studies involving human research participants and Sex and Gender in Research.

Reporting on sex and gender	Based on how a patient is listed in the electronic patient record, we used the terms male and female (table 1).
Population characteristics	See table 1.
Recruitment	Patients weren't recruited, we used retrospectively data of 3 general practices (see inclusion criteria).
Ethics oversight	The procedure of collecting retrospective data complies with all dutch privacy legislation.

Note that full information on the approval of the study protocol must also be provided in the manuscript.

Field-specific reporting

Please select the one below that is the best fit for your research. If you are not sure, read the appropriate sections before making your selection.

☐ Life sciences ☐ Behavioural & social sciences ☒ Ecological, evolutionary & environmental sciences

For a reference copy of the document with all sections, see nature.com/documents/nr-reporting-summary-flat.pdf

Ecological, evolutionary & environmental sciences study design

All studies must disclose on these points even when the disclosure is negative.

Study description	Retrospective longitudinal study exploring trends in timing of frequent seasonal allergic rhinitis (SAR) presentation to general practitioners over 25 years and explored association with air temperature.
Research sample	All SAR patients and their GP-encounters, classified with ICPC code "allergic rhinitis" or "allergic conjunctivitis and exclusion of the ICD-10 subcoding 'other specified allergic rhinitis" to include as many patients with season allergic rhinitis but also exclude patients with other allergies (dogs, cats, house dust mites).
Sampling strategy	Sampling strategy: to include as much SAR patients as possible within our data base. The general practices included in the database must had data over the whole study period to avoid bias.
Data collection	Registration of data on diagnosis and contact frequency for SAR occurs within the electronic health record (EHR) in the general practice. Registering practices are affiliated to the practice based research network (PBRN) which is a collaboration between the practices and the Radboud University Medical Centre. All data registered for the PBRN are extracted from the EHR and stored de-identified in the Radboudumc Technology Center (RTC) Health Data. RTC processes and secures the data for research purposes and secures privacy of involved patients. Listed patients in the practices may opt-out for extraction of their data. This procedure complies with all Dutch privacy legislation.
Timing and spatial scale	Data were collected retrospectively from a database with already existing data concerning the period 1995 untill 2019.
Data exclusions	See above.
Reproducibility	Our study is not a classic experiment, we did not recruite data for this specific goal but used already existed data. We defined our methods and definitions so carefully as possible, to make it as easy as possible to reproduce our researchmethod (retrospective as wel prospective)
Randomization	This is not relevant to our study, because we didn't not make groups before the results. We made group based on the results (cold, warm, intermediate) and therefor not randomized.

Blinding	Blinding wasn't possible because it's a retrospective study. The general practitioners participating in the fame-net database are well trained to document data for the database as good as possible.

Did the study involve field work? ☐ Yes ☒ No

Reporting for specific materials, systems and methods

We require information from authors about some types of materials, experimental systems and methods used in many studies. Here, indicate whether each material, system or method listed is relevant to your study. If you are not sure if a list item applies to your research, read the appropriate section before selecting a response.

Materials & experimental systems

n/a	Involved in the study
☒	☐ Antibodies
☒	☐ Eukaryotic cell lines
☒	☐ Palaeontology and archaeology
☒	☐ Animals and other organisms
☒	☐ Clinical data
☒	☐ Dual use research of concern

Methods

n/a	Involved in the study
☒	☐ ChIP-seq
☒	☐ Flow cytometry
☒	☐ MRI-based neuroimaging
